# Estimating Cardiovascular Benefits of Tirzepatide in Sleep Apnea and Obesity: Insight from the SURMOUNT-OSA Trials

**DOI:** 10.1007/s13679-024-00592-x

**Published:** 2024-10-08

**Authors:** Guglielmo Beccuti, Fabio Bioletto, Mirko Parasiliti-Caprino, Andrea Benso, Ezio Ghigo, Alessandro Cicolin, Fabio Broglio

**Affiliations:** 1 Department of Medical Sciences, University of Turin, Corso Dogliotti 14, 10126 Turin, Italy; 2 Department of Neurosciences “Rita Levi Montalcini”, University of Turin, Turin, Italy

**Keywords:** Tirzepatide, Sleep apnea, Obesity, Apnea–hypopnea index, Hypoxic burden, Cardiovascular risk

## Abstract

**Purpose of Commentary:**

This commentary aims to offer a perspective on the effect of tirzepatide on hypoxic burden and provide indirect evidence of cardiovascular risk reduction after tirzepatide for the treatment of obstructive sleep apnea and obesity. It also discusses the role of tirzepatide-induced weight loss in the management of obstructive sleep apnea.

Recent Findings.

In the SURMOUNT-OSA phase 3 trials, tirzepatide, a new GIP/GLP-1 receptor co-agonist, reduced the apnea–hypopnea index, hypoxic burden, and body weight in adults with moderate-to-severe obstructive sleep apnea and obesity. The change in apnea–hypopnea index is clinically relevant, but its impact on cardiovascular mortality remains unclear. Conversely, hypoxic burden predicts cardiovascular mortality across populations independent of AHI.

**Summary:**

We attempted to postulate the magnitude of cardiovascular benefits of tirzepatide based on the reduction in hypoxic burden. Tirzepatide treatment for obstructive sleep apnea and obesity seems to result in hypoxic burden values associated with a lower cardiovascular mortality rate and thus might attenuate the negative cardiovascular impact of hypoxic burden.

## Introduction

Malhotra and Collaborators have published the results of the SURMOUNT-OSA phase 3 trials [1], destined to be a milestone in the multidisciplinary management of obstructive sleep apnea (OSA) and obesity. Adults with moderate-to-severe OSA (apnea–hypopnea index—AHI ≥ 15 events per hour) and obesity (body mass index—BMI ≥ 30 or ≥ 27 kg/m^2^ in Japan) were enrolled in two trials. Trial 1 included those not receiving positive airway pressure (PAP) treatment at baseline, and trial 2 included those receiving stable PAP therapy, with a 7-day PAP washout period at baseline, week 20, and week 52 assessment. Participants were randomized to receive either tirzepatide (10 or 15 mg), the first GIP/GLP-1 receptor co-agonist, or a placebo. Tirzepatide reduced the AHI, hypoxic burden, and body weight and improved sleep-related patient-reported outcomes in both trials.

Professor Patel has raised some questions [2], particularly about AHI as a surrogate marker and the potential cardiovascular benefits of tirzepatide, which are yet to be demonstrated in this clinical context.

Herein, we briefly discuss the weight loss effect of tirzepatide in OSA management, polysomnographic parameters other than AHI, and the indirect evidence of cardiovascular risk reduction after tirzepatide treatment. We also debate the role of PAP treatment in the light of the above updates.

## Tirzepatide-induced Weight Loss in OSA Management

The American Heart Association Scientific Statement on OSA and cardiovascular disease affirms that the risk of OSA correlates with BMI, and obesity remains the one major modifiable risk factor for OSA [3]. This Statement refers to a population-based cohort study demonstrating that 10% weight gain is associated with a 32% increase in AHI, and even modest weight control effectively reduces new OSA onset or AHI severity [4]. A recent meta-analysis showed that, regardless of the intervention used, a weight or BMI reduction of 10%, 20%, and 30% is associated with a 36%, 57%, and 69% decrease in AHI, respectively [5]. For these reasons, behavioral modifications and weight loss interventions should be considered for all OSA patients [3].

In the SURMOUNT trial 1, the estimated treatment difference (ETD) in weight was -16.1% (95% confidence interval -18.0, -14.2), and the ETD in AHI was -47.7% (-65.8, -29.6). Similarly, in trial 2, the ETD in weight was -17.3% (-19.3, -15.3), and the ETD in AHI was -56.2% (-73.7, -38.7), close to that expected with a 20% weight loss [5]. It remains unclear whether the AHI change was dependent on weight loss only, so exploring the relationship between the AHI improvement degree and the weight reduction magnitude would be compelling.

Although a cross-sectional study observed a non-linear relationship between AHI and BMI characterized by almost steady values of AHI as BMI increases up to 35 kg/m^2^ [6], the meta-analysis by Malhotra et al. included weight loss studies in OSA with baseline BMI < 35 mg/m^2^ that demonstrate clear dose–response reductions in AHI [5] and thus reinforce the need for all patients with obesity-associated OSA to lose excess adipose tissue.

## Polysomnographic Parameters as Predictors of Cardiovascular and All-cause Mortality

Patel has reminded us about insufficient evidence to assess the validity of AHI change as a surrogate or intermediate measure for long-term health outcomes [2]. Emerging data suggest that AHI might be the wrong target for OSA treatment, particularly when aimed at cardiovascular risk reduction, as it does not reflect the complexity of OSA [7]. Other markers have been proposed as predictors of OSA subtypes at higher risk for cardiovascular disease or all-cause mortality [7, 8]. These oximetry-derived parameters provide information about the depth and duration of respiratory event desaturations; they include different calculation methods of time below 90% saturation, as well as desaturation area-based parameters like hypoxic burden (HB), respiratory event desaturation transient area, desaturation severity, and sleep breathing impairment index [8].

For example, a longitudinal cohort analysis of the Sleep Heart Health Study found that shorter respiratory event duration predicted higher 11-year all-cause mortality, over and beyond that predicted by the AHI [9].

A retrospective monocenter study showed that oximetry-derived parameters (mainly T90) but not AHI were significant risk factors for all-cause mortality [10].

An analysis of two cohorts (Outcomes of Sleep Disorders in Older Men Study—MrOS and Sleep Heart Health Study—SHHS) demonstrated that HB predicted cardiovascular mortality [11]. The authors used a Cox model adjusted for the following covariates: AHI, two measures of overnight hypoxemia (percent sleep time < 90% saturation and average event-related minimum saturation), age, BMI, gender (SHHS), race, smoking status, alcohol use (MrOS), chronic obstructive pulmonary disease, renal failure (MrOS), hypertension, diabetes, stroke, congestive heart failure, concurrent cardiovascular diseases (MrOS: coronary heart disease, peripheral vascular disease, claudication, myocardial infarction, angina, and transient ischemic attack; SHHS: angina, myocardial infarction, and coronary revascularization), and lipid-lowering medication use. In the MrOS, the cardiovascular mortality adjusted hazard ratio (A-HR) for HB was 1.81 (1.25, 2.62) and 2.73 (1.71, 4.36) in the fourth (Q4 53–88%min/h) and fifth (Q5 > 88%min/h) quintiles, respectively. In the SHHS, the A-HR for HB was 1.96 (1.11, 4.3) in the highest quintile (Q5 > 71%min/h). When modeling HB as a log-transformed variable, the A-HR was 2.03 (1.37, 3.00) and 1.73 (1.08, 2.78) in the MrOS and SHHS, respectively.

Hypoxia parameters appear more strongly associated with negative cardiovascular outcomes or increased mortality than AHI [8, 12]. However, hypoxia alone cannot explain the OSA-related cardiovascular risk; other complex mechanisms are involved [12].

## Indirect Evidence of Tirzepatide Cardiovascular Benefits in OSA and Obesity

A prespecified, key secondary endpoint of SURMOUNT-OSA trials was the change in sleep apnea-specific HB [1]. In the tirzepatide groups, the HB change at week 52 was -95.2%min/h (-103.2, -87.2) and -100.3%min/h (-110.3, -95.6) for the trial 1 and 2 treatment-regimen estimands, respectively. It would be intriguing to know who had the most significant change in HB and whether this improvement was mediated mainly by weight loss. Some OSA endotypes do not necessarily recognize obesity as the primary etiological mechanism; conversely, adiposity-related endotypes might show a higher response to tirzepatide.

Based on the huge HB variation, we tried to calculate the impact of tirzepatide on cardiovascular risk. Mean HB at baseline (153.6 and 132.2%min/h in trials 1 and 2, respectively) would correspond to the MrOS Q5 (A-HR 2.73) and SHHS Q5 (A-HR 1.96) for both trials. After 52 weeks, tirzepatide would likely lower the mean HB to 58.4%min/h in trial 1, corresponding to the MrOS Q4 (A-HR 1.8; 1.25, 2.62) and SHHS Q4 (non-significant HR), and 31.9%min/h in trial 2, corresponding to the MrOS Q3 (non-significant HR) and SHHS Q3 (non-significant HR). Although limited by using aggregate data, these hypotheses would point towards a significantly lower cardiovascular risk in the tirzepatide groups. In trial 1, the A-HR for HB would decrease on average by 33% according to the MrSO analysis and appear like that of the lowest HB quintile according to the SHHS analysis. In trial 2, based on both MrSO and SHHS, the A-HR would not differ from that of the lowest HB quintile.

Moreover, we calculated the A-HR for log-transformed HB based on the SHHS analysis: 0.74 and 0.64 in trials 1 and 2, respectively, with a nearly 30% reduction in the cardiovascular mortality rate in the tirzepatide groups, consonant with the previous estimate.

This speculation about the reduction of cardiovascular mortality rate assumes an isolated change in HB with weight loss. However, efficacious weight loss interventions have a positive impact on BMI and other obesity-related factors like lipids and blood pressure. Tirzepatide, which represents a multi-target therapy, has shown to improve weight-related parameters, glycemic and lipid levels, blood pressure [13], and inflammatory markers [1]. Consequently, although the proposed cardiovascular mortality rate reduction is specifically estimated from the reduction in HB, this rate is likely to be underestimated because of improvement in other cardiovascular risk factors that accompany weight loss.

## Positioning Tirzepatide Once Weekly: Versus or in Addition to Daily PAP Use?

The field of sleep medicine is facing a potential change in the way OSA with co-morbid overweight/obesity is managed. On the one hand, the gold standard treatment with PAP has failed to demonstrate meaningful improvements in long-term health outcomes [14]. On the other hand, weight loss of the magnitude shown in SURMOUNT-OSA has demonstrated a reduction in nightly OSA and sleepiness that could be comparable to PAP alone, but with additional improvements in important markers of cardiometabolic risk including blood pressure. Although these results suggest that weight loss with incretins could replace PAP as first-line therapy for OSA with co-morbid overweight or obesity, direct head-to-head comparative effectiveness trials that focus on AHI reduction, sleepiness, sleep-related quality of life, and blood pressure must first be conducted to provide supporting evidence. While longer trials could also demonstrate superior reductions in hard cardiovascular endpoints, such trials are probably not feasible.

## Key References

•• Malhotra A, Grunstein RR, Fietze I, Weaver TE, Redline S, Azarbarzin A, et al. Tirzepatide for the Treatment of Obstructive Sleep Apnea and Obesity. N Engl J Med. Jun 21 2024. https://doi.org/10.1056/NEJMoa2404881
  Provides the results of SURMOUNT-OSA phase 3 trials
• Azarbarzin A, Sands SA, Stone KL, Taranto-Montemurro L, Messineo L, Terrill PI, et al. The hypoxic burden of sleep apnoea predicts cardiovascular disease-related mortality: the Osteoporotic Fractures in Men Study and the Sleep Heart Health Study. Eur Heart J. Apr 07 2019;40(14):1149-1157. https://doi.org/10.1093/eurheartj/ehy624
  Addresses the role of hypoxic burden as a predictor of cardiovascular mortality independent of AHI

## Data Availability

No datasets were generated or analysed during the current study.
